# Therapeutic effect of concentrated growth factor gel (CGF) on postoperative defects of giant skin tumors

**DOI:** 10.1097/MD.0000000000045014

**Published:** 2025-10-10

**Authors:** Binxiong Chen, Congcong Huang, Yang Liu, Bingbing Zhong, Zhuyun Zhao, Changneng Ke, Yueming Liu, Shi Xu, Songyun Zou

**Affiliations:** a Department of Burn and Plastic Surgery, Shenzhen Longhua District Central Hospital, Shenzhen, Guangdong Province, China; b Graduate School, Jinan University, Guangzhou, Guangdong Province, China.

**Keywords:** concentrated growth factor gel, giant skin tumor, postoperative defects, therapeutic effect

## Abstract

This study aimed to evaluate the effect of concentrated growth factor (CGF) gel on the repair of defects following the resection of giant skin tumors. Data were collected from patients who utilized CGF gel to fill the defects between January 2020 and June 2024, including records of drainage fluid, pain scores, time of drainage tube removal, discharge time, healing time, infection rates, and patient satisfaction. Data were analyzed using SPSS version 27.0. 58 patients were included in the study, comprising 35 males and 23 females. There were 32 patients in Group A (CGF with drainage) and 26 patients in Group B (drainage only). The postoperative healing time (11.5 ± 1.55 days), hospitalization duration (7.31 ± 0.90 days), and drainage tube removal time (4.03 ± 0.97 days) in Group A were significantly shorter than those in Group B. Additionally, the drainage volume on the third day (11.38 ± 2.51 mL) and the fifth day (1.42 ± 3.83 mL) postoperation were also lower in Group A compared to Group B (*P* < .05). The use of CGF gel significantly shortened healing and hospitalization times, reduced drainage fluid volume, and improved patients’ satisfaction. Compared to conventional surgery, the application of CGF on postoperative defects of giant skin tumors deserves clinical promotion and popularization.

## 1. Introduction

Clinically, giant skin tumors, including shoulder and neck fat pads, huge back lipomas, and epidermoid cysts, are more common. Surgical resection is currently a widely used treatment method. After surgery, patients may experience significant tissue defects and a large postoperative cavity. These issues can lead to long-term complications such as cavity formation, fat liquefaction, effusion, infection, and incision cracking. These complications can increase pain and burden on the patient, as well as pose challenges for clinicians. Concentrated growth factor (CGF) is the latest generation of platelet-rich, autologous blood concentrate products.^[1]^ Studies have shown that it promotes tissue regeneration, wound healing and infection control.^[2]^ Kao CH observed 16 patients treated with CGF and found that they had obvious granulation tissue and regenerated epidermis during the healing process, confirming the significant effect of CGF treatment in promoting overall wound healing.^[3]^ Our previous study demonstrated CGF could promote wound healing potential of HaCaT cells by activating the RAS signaling pathway.^[4]^ However, no relevant reports of CGF being used for the treatment of postoperative defects of huge skin tumors were found. After resection of giant skin tumors, CGF gel was used to fill the postoperative defects and cavity, resulting in satisfactory treatment outcomes.

## 2. Materials and methods

### 2.1. Study design

The clinical data of patients with giant skin tumors who visited the Burn and Plastic Surgery Department of our hospital between January 2020 and June 2024 were reviewed and analyzed. This study was reviewed and approved by the Medical Ethics Committee of Shenzhen Longhua District Central Hospital (No.: 2024-015). Given the retrospective nature of the study and the use of de-identified patient data, the requirement for individual informed consent was waived by the Ethics Committee. All research procedures adhered to the ethical standards of the Helsinki Declaration and the relevant national and international guidelines for medical research involving human subjects.

The patients were divided into 2 groups: group A received CGF gel treatment in addition to routine surgical treatment, whereas group B received only regular surgical treatment. The inclusion criterion for the study was patients with giant skin tumors that resulted in tissue defects following surgical resection (>25 cm^2^). The exclusion criteria were as follows: serious diseases of important organs such as the heart, lung, and brain; serious diseases that affect healing, such as diabetes, chronic kidney disease, immune diseases, and malnutrition; and incomplete information.

### 2.2. Treatment method

CGF gel preparation: In the morning, on an empty stomach, the patient’s blood was drawn intravenously and collected using a sterile vacuum tube without an anticoagulant (manufactured by Grena, Austria). Subsequently, the blood was put into a special CGF centrifuge (Medifuge Centrifuge System Silfradentsrl, Sofia, Italy, Sefadente) and centrifuged according to the following steps: accelerate to 2700 rpm for 2 minutes, hold at 2400 rpm for 4 minutes, then at 2700 rpm for 4 minutes, followed by 3000 rpm for 3 minutes, and finally slow down and stop for 36 seconds. Approximately 14 minutes was required for the entire process. Following centrifugation, the blood is separated into 3 layers: the top layer consists of serum, the middle layer contains CGF fibrin gel, and the bottom layer consists of red blood cells and platelets. The middle layer of CGF fibrin gel was taken for later use.

**Figure 1.**
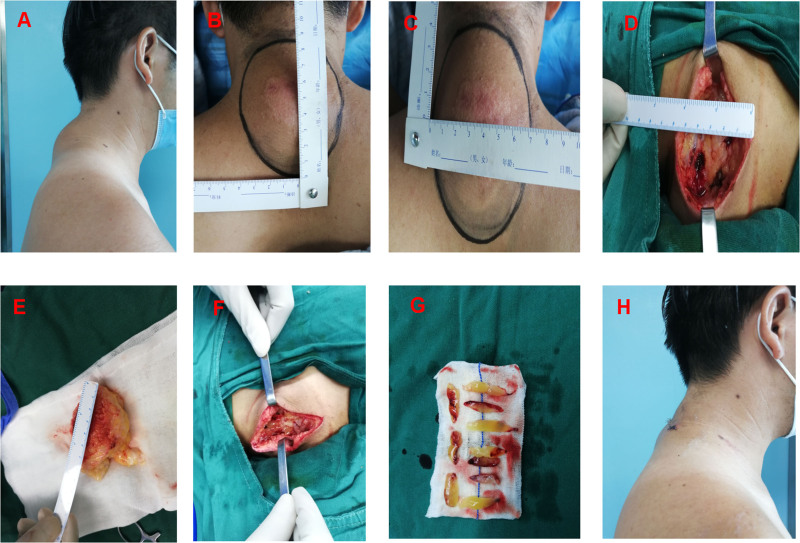
A 45-yr old male patient was treated with concentrated growth factor gel to fill the wound cavity after resection of a huge tumor in the shoulder and neck. (A) Preoperative; (B and C) ultrasound localization, marking of tumor size and resection range; (D) residual wound cavity after tumor resection; (E) excised tumor; (F) intraoperative concentrated growth factor gel was used to fill the wound cavity; (G) concentrated growth factor gel; (H) 7 days after surgery.

Surgical steps: The affected skin profile of patient on admission was recorded (Fig. 1A). Before the operation, determine the boundary of the skin tumor through color ultrasound, mark the size and resection range of the tumor (Fig. 1B and C), select local anesthesia or general anesthesia, remove larger skin tumors, and retain the wound cavity (Fig. 1D and E). After resection, Group A used CGF gel to fill the defect (Fig. 1F and G), placed a drainage tube, utilized 3-0 absorbable sutures to suture the subcutaneous fat layer, and employed 5-0 absorbable sutures to suture the epidermal layer; Group B directly placed the drainage tube after resection, used 3-0 absorbable sutures to suture the subcutaneous fat layer, and employed 5-0 absorbable sutures to suture the epidermal layer. After the operation, ice was applied for 3 days, and the incision dressing was changed once a day to record the patient’s drainage fluid changes, drainage tube removal time, and pain score. The skin profile of patient at 7-day follow-up was recorded (Fig. 1H).

### 2.3. Observation indicators

We evaluated the changes in drainage fluids, drainage tube removal time, hospitalization duration, healing time, infection incidence, pain score, and patient satisfaction in the 2 groups.

### 2.4. Statistical analysis

Meteorological data are presented as (mean ± SD), and count data are expressed as a percentage (%). The SPSS 27.0 (SPSS, Chicago) was utilized for analyzing the data, and the age, VAS score, and other measurement data were displayed as mean ± standard deviation (x ± sd). The independent sample *t* test or paired sample *t* test was performed between groups or within groups. The Chi-squared test was performed to enhance comparisons between groups. The satisfaction value was grade data, and a rank sum test was performed between groups. The *P* value < .05 was considered statistically significant.

## 3. Results

### 3.1. Demographic characteristics

This study included a total of 58 patients who underwent surgical removal of giant skin tumors and experienced postoperative defects, comprising 35 males and 23 females. Group A consisted of 32 patients with an average age of 46.5 years, whereas Group B included 26 patients with an average age of 44.96 years. The average glycated hemoglobin level in group A was 5.08% and 4.62% in group B (*P* < .01). The average fasting blood glucose level in group A was 4.98 mmol/L, while that in group B it was 4.83 mmol/L. The level of alanine aminotransferase (ALT) in group A was 22.28 U/L, and in group B it was 20.35 U/L. The creatinine level in group A was 70.37 μmol/L, and in group B it was 66.85 μmol/L. Statistical analysis showed *P* > .05, indicating no significant difference between the 2 groups (Table 1).

**Table 1:** Demographic characteristics of patients in A and B groups.

	Group A (Mean ± SD)	Group B (Mean ± SD)	*t*/χ^2^/*z* value	*P* value
Number of observations	32	26	–	–
Gender (M/F)	19/13	16/10	0.028	.867
Age (yr)	46.5 ± 7.59	44.96 ± 6.71	−.665	.506
Defect sites, [n, (%)]	Shoulder (21, 65%)	Shoulder (17, 65%)	2.837	.242
	Neck (5, 16%)	Neck (6, 23%)		
	Back (6, 19%)	Back (3, 12%)		
Blood glucose (mmol/L)	4.98 ± 0.50	4.83 ± 0.47	.28	.867
Alanine aminotransferase (U/L)	22.28 ± 9.69	20.35 ± 11.59	−.665	.506
Creatinine (μmol/L)	70.37 ± 14.69	66.85 ± 17.25	−.891	.373

### 3.2. Changes in drainage fluid

**Figure 2.**
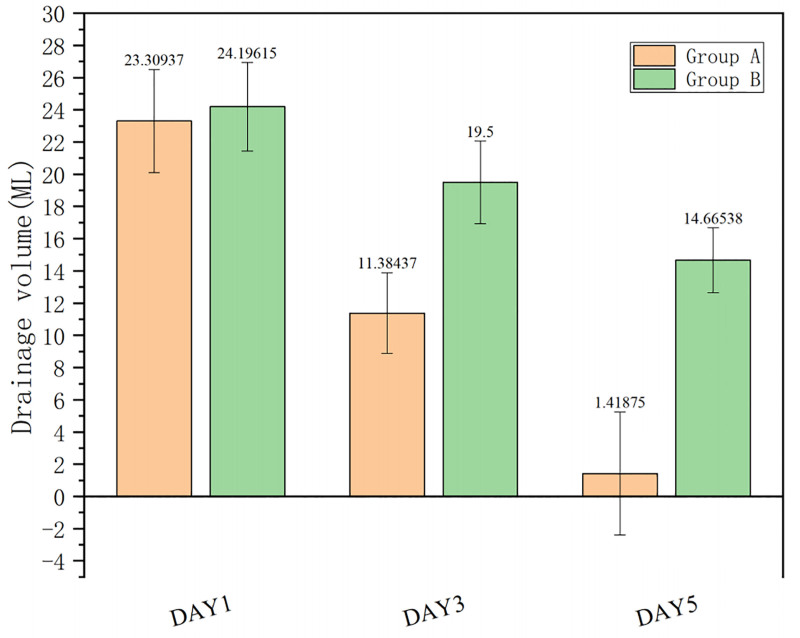
The amount of drainage in group A on the 3rd and 5th days after operation was significantly lower than that in group B (*P* < .01, Duncan post hoc test was used for differences between pairs of means).

The changes in the incision drainage volume during the postoperative period showed that there was no significant difference in the drainage volume between the 2 groups on the first day (*P* > .05). The drainage volumes of the 2 groups showed a downward trend. The drainage volume of group A was 11.38 ± 2.51 mL on the third day after surgery, and 19.5 ± 2.56 mL in group B. On the fifth day after surgery, the average drainage volume of group A was 1.42 ± 3.83 mL, and in group B, it was 14.67 ± 2.02 mL. The drainage volume of group A was more pronounced on the third and fifth days after surgery (*P* < .01) (Fig. 2 and Table S1, Supplemental Digital Content, https://links.lww.com/MD/Q236).

### 3.3. Drainage tube removal time

**Figure 3.**
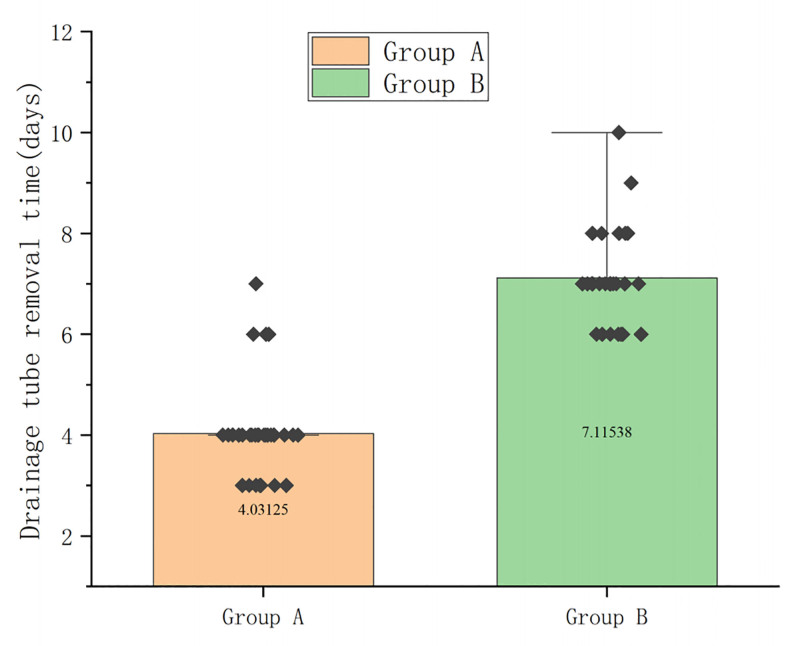
Compared with the drainage tube removal time, group A was earlier than group B, *P* < .01 (Student *t* test was used for comparison of 2 groups).

The drainage tube can be removed when the drainage liquid volume is ≤ 10 mL, as per the standard. Comparison of drainage tube removal times in the 2 groups of cases showed that the time of drainage tube removal in group A was 4.03 ± 0.97 days and the time in group B was 7.12 ± 0.99 days. The extubation time in group A was significantly shorter than that in group B (*P* < .05) (Fig. 3 and Table S2, Supplemental Digital Content, https://links.lww.com/MD/Q236).

### 3.4. Hospitalization duration

**Figure 4.**
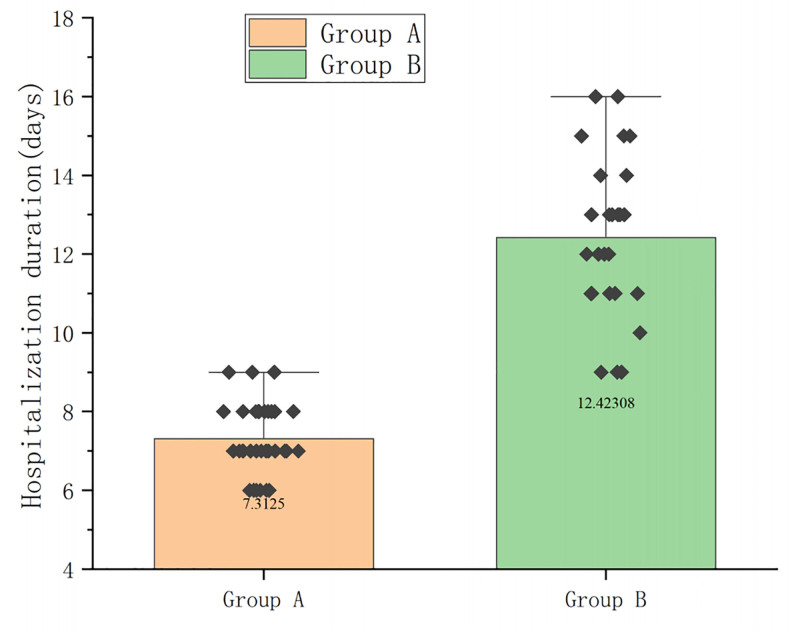
Comparing the hospitalization duration of the 2 groups, group A was significantly shorter than group B, *P* < .01 (Student *t* test was used for comparison of 2 groups).

When comparing the hospitalization duration in the 2 groups, it was found that group A had an average of 7.31 ± 0.90 days, while group B had an average of 12.42 ± 2.04 days. The hospitalization duration in group A was significantly shorter than that in group B (*P* < .01) (Fig. 4 and Table S3, Supplemental Digital Content, https://links.lww.com/MD/Q236).

### 3.5. Healing time

**Figure 5.**
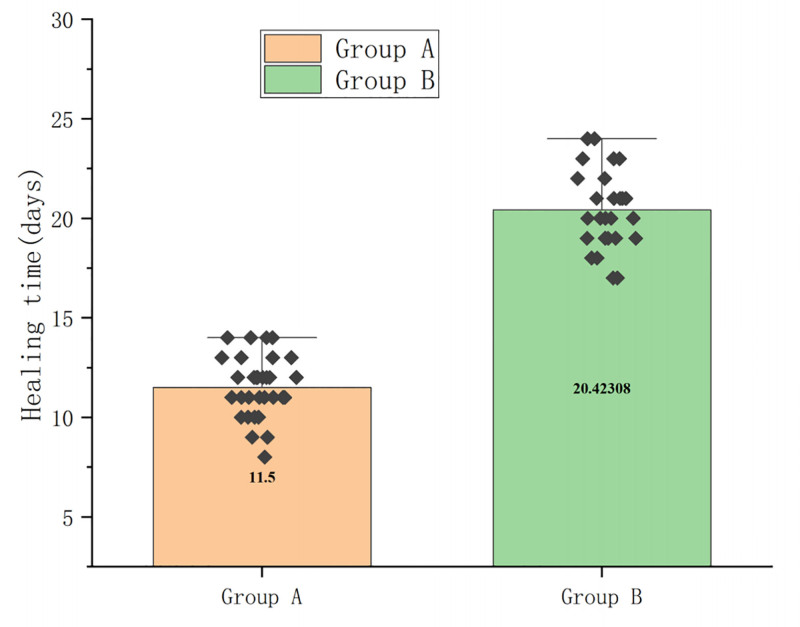
The healing time of group A was significantly lower than that of group B, *P* < .01 (Student *t* test was used for comparison of 2 groups).

According to the clinical symptoms of the incision, if there are no symptoms such as redness, swelling, effusion, and tenderness, it is believed that the incision has healed. The healing time of the 2 groups was compared, with group A having a hospital stay and outpatient time of 11.5 ± 1.55 days and group B having 20.42 ± 1.98 days. There were significant differences between the 2 groups, with the healing time in group A being significantly shorter than that in group B (*P* < .01) (Fig. 5 and Table S4, Supplemental Digital Content, https://links.lww.com/MD/Q236).

### 3.6. Postoperative infection

During the incision healing period, bacterial cultures are performed on the incision secretions every week, and if the culture result is positive, a postoperative infection is diagnosed. The incidence rate in group A was 0/32 and in group B was 2/26. When comparing the incidence of postoperative infection between the 2 groups, the incidence in Group B was higher, but the difference was not statistically significant (*P* > 0. 05) (Table 2).

**Table 2:** Analysis of incidence of infection in the A and B group.

	Infectious	Non-infectious
Group A	0	32
Group B	2	24
χ^2^	2.547	
*P*-value	.505	

### 3.7. Postoperative pain assessment

**Figure 6.**
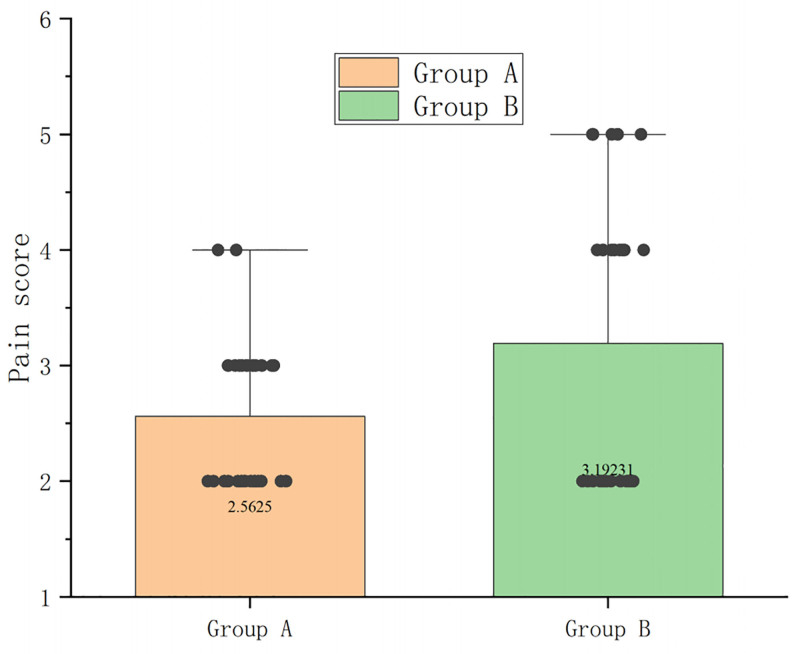
The degree of pain in group A was significantly lower than that in group B, and the difference between the 2 groups was statistically significant (*P* < .01).

The visual analog scale (VAS) was used to evaluate the intensity of pain within 24 hours after the operation; on a scale of 0 to 10, where 0 represents painless and 10 represents severe and unbearable pain, the pain score in group A was 2.56 ± 0.62, and in group B it was 3.19 ± 1.27. The pain level in Group A was significantly lower than that in Group B, with a statistically significant difference between the 2 groups (*P* < .05) (Fig. 6 and Table S5, Supplemental Digital Content, https://links.lww.com/MD/Q236).

## 4. Discussion

After the surgical removal of large skin tumors, promoting wound healing, reducing complications, and enhancing patient experience remain critical concerns in clinical practice. Clinicians typically accelerate healing by effectively closing dead spaces, thoroughly stopping bleeding, ensuring appropriate drainage, and applying postoperative compressions. Recently, CGF gel has gained widespread use in facial rejuvenation, acne scar repair, bone defect repair, periodontal tissue regeneration, postoperative jaw filling, and ulcerative wounds.^[5–10]^ As a concentrated product of autologous blood, CGF is enriched with growth factors, fibrin, and leukocytes, and features a highly biocompatible 3-dimensional fibrous network structure. We conducted a retrospective analysis of 58 patients who underwent surgical removal of large skin tumors to evaluate the effectiveness of CGF gel for treating severe defects.

Our findings demonstrated that the CGF group had significantly lower drainage volumes on postoperative days 3 and 5 than the non-CGF group, and the time to tube removal was shorter, indicating that CGF gel effectively reduced wound exudate. This may be attributed to the 3-dimensional fibrin network in CGF, which can directly cover the wound and form a semipermeable membrane, thus reducing tissue fluid exudation.^[11–13]^ Simultaneously, CGF can inhibit pro-inflammatory factors such as IL-6 and TNF-α, up-regulate anti-inflammatory factors such as IL-10, reduce vascular endothelial cell damage, and further decrease vascular permeability.^[4,14]^ Additionally, platelets in CGF are partially activated during centrifugation, releasing coagulation factors that accelerate fibrin clot formation.^[15–18]^

In a clinical trial involving 60 subjects conducted by Özveri Koyuncu B et al., CGF showed better soft tissue healing after mandibular third molar extraction compared to the non-CGF group.^[19]^ The randomized controlled trial from Amato *et al* provided solid evidence for CGF use in leg ulcers,^[20]^ which was consistent with previous study.^[21]^ Our study results indicated that the CGF group had a significantly shorter hospital stay and healing period than the non-CGF group, consistent with previous studies of therapeutic effect of CGF on postoperative wound healing.^[19]^ The mechanisms include CGF promotes the proliferation and migration of fibroblasts and keratinocytes;^[4,14]^ CGF regulates angiogenesis through both direct induction and paracrine signaling;^[11,22]^ CGF up-regulates type I collagen and fibronectin, increases extracellular matrix deposition, and inhibits MMP-2 activity, reducing matrix degradation;^[14,23]^ CGF promotes tissue regeneration through immunomodulation and the recruitment and activation of stem cells.^[24,25]^

By comparing the 2 groups of patients, we found that the VAS score in the CGF group was significantly lower than that in the non-CGF group at 24 hours postoperatively, indicating that CGF helps relieve postoperative pain, which was consistent with previous studies.^[10,19]^ This effect may be due to TGF-β in CGF, which reduces the expression of inflammatory mediators, such as IL-6 and TNF-α, thereby decreasing nociceptive sensitivity.^[26]^ Furthermore, PDGF and VEGF promote the proliferation and axonal regeneration of Schwann cells.^[27]^

Relatively little research has been conducted on the antibacterial activity of platelet concentrates. Some studies have shown that autologous platelet concentrate can inhibit microorganism growth by acting bacteriostatically rather than bactericidally.^[28]^ However, antibacterial peptides released by platelets and leukocytes in CGF, such as β-defensins and LL-37, exert a bactericidal effect by disrupting bacterial cell membranes, inhibiting cell wall synthesis, or interfering with DNA replication. In vitro experiments demonstrated that CGF had an antibacterial effect on Aggregatibacter actinomycetemcomitans and Porphyromonas gingivalis.^[29]^A study by Alauddin et al also found that CGF was effective against Staphylococcus aureus and Streptococcus mutans.^[30]^ In our study, the infection rate in the non-CGF group (2/26) was higher than that in the CGF group (0/32); however, the difference was not significant, likely due to the small sample size.

Our study demonstrated that the treatment group receiving CGF gel exhibited significantly better recovery outcomes than the control group. The CGF group reported higher satisfaction scores (Table S6, Supplemental Digital Content, https://links.lww.com/MD/Q236), which was likely attributed to a shorter healing duration and reduced postoperative pain. However, several limitations still exist in our study. As main aim of this study is to observe the therapeutic effect of CGF on wound cavity repair, the follow-up study was missed. Scar quality has crucial influence on patients’ experience, however Vancouver Scar Scale assessment was terminated due to data loss. Besides, this study’s retrospective case-control design and small sample size limit the findings, as the long-term effects and underlying mechanisms were not thoroughly investigated. In future study, we will explore the role of CGF in the current landscape of surgical strategies used to obliterate dead space compared to other strategies, such as closed-suction drains, fibrin sealants/glues, quilting sutures, barbed sutures, or compression garments, which are all commonly used tools in the surgeon’s armamentarium.

In summary, our research indicates that CGF is effective in treating severe defects following the surgical removal of skin tumors. Patients in the CGF group experienced a shorter healing period, less postoperative discomfort, and overall improved patient satisfaction compared to those in the non-CGF group.

## Acknowledgments

We are grateful to all study participants for their participation in the study.

## Author contributions

**Conceptualization:** Binxiong Chen, Songyun Zou.

**Data curation:** Bingbing Zhong, Changneng Ke.

**Formal analysis:** Congcong Huang, Yang Liu, Yueming Liu.

**Funding acquisition:** Yueming Liu, Shi Xu.

**Investigation:** Bingbing Zhong.

**Methodology:** Changneng Ke, Shi Xu, Songyun Zou.

**Project administration:** Yang Liu, Zhuyun Zhao.

**Resources:** Congcong Huang, Yang Liu, Yueming Liu.

**Software:** Bingbing Zhong.

**Supervision:** Zhuyun Zhao, Yueming Liu.

**Validation:** Zhuyun Zhao.

**Visualization:** Zhuyun Zhao.

**Writing – original draft:** Binxiong Chen.

**Writing – review & editing:** Binxiong Chen, Shi Xu, Songyun Zou.

## Supplementary Material


