# Supplementary material for: Therapeutic effect of concentrated growth factor gel (CGF) on postoperative defects of giant skin tumors
Source: Medicine (Baltimore). 2025 Oct 10;104(41):e45014. doi: 10.1097/MD.0000000000045014 (PMC12517886; doi:10.1097/MD.0000000000045014)
Supplement: Supplementary file 1 [file medi-104-e45014-s001.docx]

**Table S1** Analysis of drainage volume on day-1、day-3 and day-5 in group A and B

|  | DAY-1(Mean±SD) | DAY-3(Mean±SD) | DAY-5(Mean±SD) |
| --- | --- | --- | --- |
| Group A(ml) | 23.30±3.20 | 11.38±2.51 | 1.42±3.83 |
| Group B(ml) | 24.20±2.75 | 19.5±2.56 | 14.67±2.02 |
| T | -1.12 | -12.14 | -13.25 |
| **p-value** | 0.27 | <0.01 | <0.01 |

**Table S2** Analysis of drainage tube removal time in group A and B

|  | Drainage tube removal time(Mean±SD) |
| --- | --- |
| Group A(days) | 4.03+0.97 |
| Group B(days) | 7.12+0.99 |
| T | 11.94 |
| **p-value** | <0.01 |

**Table S3** Analysis of hospitalization duration in group A and B

|  | Hospitalization duration(Mean±SD) |
| --- | --- |
| Group A(days) | 7.31±0.90 |
| Group B(days) | 12.42±2.04 |
| T | -12.74 |
| **p-value** | <0.01 |

**Table S4** Analysis of healing time in group A and B

|  | Healing time(Mean±SD) |
| --- | --- |
| Group A(days) | 11.5±1.55 |
| Group B(days) | 20.42±1.98 |
| T | -19.26 |
| **p-value** | <0.01 |

**Table S5** Analysis of pain score in group A and B

|  | Pain score(Mean±SD) |
| --- | --- |
| Group A | 2.56±0.62 |
| Group B | 3.19±1.27 |
| T | -2.48 |
| **p-value** | <0.01 |

**Table S6** Analysis of patient satisfaction in group A and B

|  | Patient satisfaction(Mean±SD) |
| --- | --- |
| Group A | 3.47±0.51 |
| Group B | 2.61±1.13 |
| T | 3.82 |
| **p-value** | <0.01 |
